# MiR-17-5p Impairs Trafficking of H-ERG K^+^ Channel Protein by Targeting Multiple ER Stress-Related Chaperones during Chronic Oxidative Stress

**DOI:** 10.1371/journal.pone.0084984

**Published:** 2013-12-30

**Authors:** Qi Wang, Weina Hu, Mingming Lei, Yong Wang, Bing Yan, Jun Liu, Ren Zhang, Yuanzhe Jin

**Affiliations:** The Fourth Affiliated Hospital of China Medical University, Shenyang, Liaoning Province, P. R. China; University of Barcelona, Spain

## Abstract

**Background:**

To investigate if microRNAs (miRNAs) play a role in regulating h-ERG trafficking in the setting of chronic oxidative stress as a common deleterious factor for many cardiac disorders.

**Methods:**

We treated neonatal rat ventricular myocytes and HEK293 cells with stable expression of h-ERG with H_2_O_2_ for 12 h and 48 h. Expression of miR-17-5p seed miRNAs was quantified by real-time RT-PCR. Protein levels of chaperones and h-ERG trafficking were measured by Western blot analysis. Luciferase reporter gene assay was used to study miRNA and target interactions. Whole-cell patch-clamp techniques were employed to record h-ERG K^+^ current.

**Results:**

H-ERG trafficking was impaired by H_2_O_2_ after 48 h treatment, accompanied by reciprocal changes of expression between miR-17-5p seed miRNAs and several chaperones (Hsp70, Hsc70, CANX, and Golga2), with the former upregulated and the latter downregulated. We established these chaperones as targets for miR-17-5p. Application miR-17-5p inhibitor rescued H_2_O_2_-induced impairment of h-ERG trafficking. Upregulation of endogenous by H_2_O_2_ or forced miR-17-5p expression either reduced h-ERG current. Sequestration of AP1 by its decoy molecule eliminated the upregulation of miR-17-5p, and ameliorated impairment of h-ERG trafficking.

**Conclusions:**

Collectively, deregulation of the miR-17-5p seed family miRNAs can cause severe impairment of h-ERG trafficking through targeting multiple ER stress-related chaperones, and activation of AP1 likely accounts for the deleterious upregulation of these miRNAs, in the setting of prolonged duration of oxidative stress. These findings revealed the role of miRNAs in h-ERG trafficking, which may contribute to the cardiac electrical disturbances associated with oxidative stress.

## Introduction

Human ether-a-go-go-related gene (h-ERG) encodes the pore-forming α-subunit of a K^+^ channel for rapid delayed rectifier current (*I*_Kr_), the major repolarizing current in cardiac cells. Dysfunction of *I*_Kr_ causes long QT syndrome (LQT), a cardiac electrical disorder that predisposes affected individuals to fatal arrhythmias and sudden death, being the major cause of familial long QT syndrome 2 (LQT2) and acquired (drug-induced) LQT [1-3]. Expression and function of h-ERG are dynamically regulated and sensitive to a wide variety of extra- and intra-cellular cues. It has been documented by a number of studies that oxidative stress results in defective function of h-ERG channel protein [4-8]. However, how oxidative stress impairs h-ERG function remained incompletely understood. 

Defective trafficking of h-ERG protein to the cell surface is the most common mechanism of h-ERG channel dysfunction in genetic LQT2 mutations and drug-induced LQT [2,9,10]. H-ERG is synthesized in the endoplasmic reticulum (ER) as an "immature" N-linked glycoprotein and is terminally glycosylated in the Golgi apparatus, and has been shown to be associated with an array of proteins including several of the cytosolic and membrane-integrated chaperones and co-chaperones, such as Hsc70 (70-kDa heat shock cognate protein), Hsp90 (90-kDa heat shock protein), Hdj-2, Hop (Hsp-organizing protein), Bag-2 (BCL-associated athanogene 2), calnexin (CANX), and FKBP38 (38-kDa FK506-binding protein, FKBP8) [11]. These chaperones are crucial for protein maturation and trafficking following synthesis, such as N-linked glycosylation of membrane proteins like h-ERG channels [12-18]. Oxidative stress is known to be one of the most frequent triggers of ER perturbation [19-21], which results in ER stress via the activation of complex cytoplasmic and nuclear signaling pathways, collectively termed the unfolded protein response [22-24]. ER stress plays fundamental roles in the development and progression of cardiovascular diseases, including heart failure, ischemic heart diseases, diabetic cardiomyopathy, atherosclerosis, and stroke. ER stress is initially an adaptive response with increased expression of chaperones to ensure correct protein folding, inhibition of protein synthesis to prevent accumulation of misfolded proteins, and degradation of misfolded proteins. After prolonged period, however, ER stress becomes detrimental by turning on the apoptotic program. Central to ER stress is the upregulation of glucose-regulated protein 78 (GRP78) belonging to the heat shock protein 70 family, along with an array of chaperones and co-chaperones [25,26]. These alterations can affect not only the cellular function and even cell fate but also the trafficking and maturation of newly synthesized proteins. 

On the other hand, microRNAs (miRNAs) have been documented to be the key regulators of protein-coding genes over the past years and their role in regulating ion channel-coding genes in the cardiovascular system have also been unraveled by a number of studies [27-32]. While miRNAs are well known to primarily act as repressors of ion channel gene expression at the post-transcriptional level, whether they also regulate trafficking of proteins remains unknown. In lieu of the facts that miRNAs are connected to ER stress signaling [33] and many of the ER stress-related chaperones and co-chaperones are potential target genes for miRNAs, it is quite plausible that miRNAs may be involved in regulating protein trafficking. Furthermore, it is also conceivable that h-ERG trafficking is regulated by miRNAs through targeting chaperones as oxidative stress-induced ER stress is associated aberrant alterations of chaperones. The primary objective of this study was to examine this hypothesis. 

## Methods

### Myocyte Isolation and Primary Cell Culture

Single ventricular myocytes from neonatal rats (NRVMs) were isolated by enzymatic dispersion techniques following the same procedures as described in detail elsewhere [20]. In brief, rats of 1-3 days old were decapitated and their hearts were aseptically removed. The atria were dissected, minced and trypsinized at 37°C for 10 min. Dispersed NRVMs were plated in 24-well plates in Dulbecco’s Modified Eagle Medium (DMEM, Invitrogen) containing 10% fetal bovine serum and 0.1 mM bromodeoxyuridine (Sigma) to inhibit the growth of fibroblasts. The non-adherent NRVMs were removed and seeded in a 24-well plate at 1×10^5^/well for further experiments. The NRVMs were verified by positive staining with an anti-β-actin monoclonal antibody through immunocytochemistry. All procedures involving animal use were approved by the Animal Care and Use Committee of the China Medical University. The adult rats were sacrificed by overdose anaesthesia (sodium pentobarbital) immediately after giving birth.

### Cell Culture

The HEK293 human embryonic kidney cell line was purchased from the American Type Culture Collection (Manassas, VA). The cells were grown in 5% CO_2_ at 37°C in DMEM supplemented with 10% fetal bovine serum.

### Induction of ER Stress by Oxidative Stress

To create conditions of early and late phases of ER stress, NRVMs were treated with 20 nmol/L H_2_O_2_ by incubation in culture medium for 12 h and 48 h, and HEK293 cells were incubated with 40 nmol/L H_2_O_2_ by incubation for 12 h and 48 h, respectively. 

### Quantitative Real-Time RT-PCR Analysis

Total RNA samples were isolated NRVMs with Trizol reagents (Ambion). The first-strand cDNA was synthesized by reverse transcription (RT) reactions using mirVana qRT-PCR primer sets (Ambion). SYBR green qRT-PCR was performed on a thermocycler ABI Prism® 7500 fast (Applied Biosystems) for 40 cycles. Fold variations in expression of an mRNA or a miRNA between RNA samples were calculated. U6 was used an internal control for inter-tube variations and Gapdh was used as an internal control for relative quantification. The threshold cycle (CT) is defined as the fractional cycle number at which the fluorescence passes the fixed threshold.

### Western Blot Analysis

Membrane, cytosolic, and nuclear protein samples were extracted separately from cultured NRVMs or HEK293 cells, and quantified by BCA Protein Assay Kit with bovine serum albumin as the standard. Protein samples at an amount of 80 µg each were fractionated by SDS-PAGE (10% polyacrylamide gels) and then transferred to PVDF membrane (Millipore, Bedford, MA). The samples were then incubated overnight at 4°C with the polyclonal anti-h-ERG antibody raised in rabbit against highly purified peptide (CY)EEL PAGAPELPQD GPT, corresponding to residues 1118–1133 of human ERG (Alomone Laboratories, Jerusalem, Israel), anti-GRP78/BIP (rabbit polyclonal; Sigma-Aldrich), anti-Hsp70 (mouse monoclonal; Abcam, ab47455), anti-Hsc70 (rat monoclonal; Abcam, ab19136), anti-Hsp90 (rat monoclonal; Abcam, ab13495), anti-Hsp40 (rabbit polyclonal; Abcam, ab23356), anti-CANX (Sigma-Aldrich), anti-Golga2 (Sigma-Aldrich), and anti-AP1 (rabbit polyclonal; Abcam). Next day, the membrane was washed three times (10 min/each) in TBS + Tween 20 and incubated for 2 h with the horseradish peroxidase-conjugated donkey anti-goat IgG (1:600 dilution) in the blocking buffer. Primary and secondary antibodies were purchased from Santa Cruz Biotechnology (Santa Cruz, CA). Finally, the membrane was rinsed with PBS before scanning using the Infrared Imaging System (LI-COR Biosciences). Bound antibodies were detected using the chemiluminescent substrate (Western Blot Chemiluminescence Reagent Plus, NEN Life Science Products, Boston, MA). Gapdh was used as an internal control for equal input of protein samples, using anti-Gapdh antibody (Santa Cruz Biotechnology Inc.). Immunoblot band density was quantified using QuantityOne software by measuring the band intensity (Area × OD) and normalizing to Gapdh. Relevant data are expressed as fold changes by normalizing the data to the control values.

### Synthesis of MiRNAs and Anti-miRNA Antisense Inhibitors

miR-17-5p (5’-C**AAAGUGC**UUACAGUGCAGGUAG -3’; boldface letters indicate the seed motif), mutant miR-17-5p (5’-C**AccccGC**UUACAGUGCAGGUAG -3’; lowercase letters indicate nucleotides replacement mutation), its antisense oligonucleotides inhibitor miR-17-5p-I (5’-CUACCUGCACUGUAAGCACUUUG-3’), and mutant antisense (5’-CUACCUGCACUGUAAGCggggUG-3’) were synthesized by Integrated DNA Technologies Inc (IDT). Five nucleotides or deoxynucleotides at both ends of the antisense molecules were modified with the ribose ring being constrained by a methylene bridge between the 2’-*O*- and the 4’-*C* atoms to enhance cellular stability and target affinity [34]. 

### Transfection Procedures

NRVMs and h-ERG expressing HEK293 cells were transfected with miR-17-5p (10 nmol/L), mutant miR-17-5p (10 nmol/L), miR-17-5p-I (4 nmol/L), or mutant miR-17-5p-I (4 nmol/L), with lipofectamine 2000 (Invitrogen), according to manufacturer’s instructions. Forty-eight hours after transfection, cells were collected for total miRNA isolation and protein purification.

### Construction of Chimeric MiR-17-5p-Target Site–Luciferase Reporter Vectors

To construct reporter vectors bearing miR-17-5p target sites, we synthesized (by Invitrogen) fragments containing the exact target sites for miR-17-5p in the 3’UTRs of Hsp70, Hsc70, CANX, GOLGA2, or the mutated target sites, and inserted these fragments separately into the multiple cloning sites downstream the luciferase gene (HindIII and SpeI sites) in the pMIR-REPORTTM luciferase miRNA expression reporter vector (Ambion, Inc.).

### Construction of Chimeric Promoter–Luciferase Fusion Plasmids

To construct reporter vectors bearing the promoter regions containing the putative *cis*-acting elements for AP1 of miR-17~92, miR-106a~363, and miR-106b~25 cluster genes, we PCR synthesized (by Invitrogen) fragments of 800 bp upstream the transcription start sites of these genes. The PCR products were subcloned separately into the multiple cloning sites upstream the luciferase gene in the PGL3-Basic (Promega, Madison, WI) vector.

### Luciferase Activity Assay

NRVMs and HEK293 cells in culture were transfected with luciferase vector and miR-17-5p or other constructs or AP1 decoy oligodeoxynucleotides for 48 h. Before transfection, cells were starved to synchronize growth by incubating in serum- and antibiotics-free medium for 12 h. Luciferase activities were measured with a dual luciferase reporter assay kit (Promega, Madison, WI) on a luminometer (Lumat LB9507).

### Whole-Cell Patch-Clamp Recording

H-ERG K^+^ current (I_h-ERG_) was recorded with whole-cell patch-clamp techniques in the voltage-clamp mode with an Axopatch-200B amplifier (Axon Instruments) in h-ERG-expressing HEK293 cells. The tip resistances of borosilicate glass electrodes were 1–3 MΩ when filled with the internal solution containing (in mmol) 130 KCl, 1 MgCl_2_, 5 Mg-ATP, 10 EGTA, and 10 HEPES (pH 7.3). The Tyrode extracellular solution contained (in mmol) 136 NaCl, 5.4 KCl, 1 CaCl_2_, 1 MgCl_2_, 10 glucose, and 10 HEPES (pH 7.4). Experiments were conducted at 36 ± 1°C. Junction potentials were zeroed before formation of the membrane-pipette seal. Series resistance and capacitance were compensated, and leak currents were subtracted.

### Statistical Methods

Group data are presented in the format of mean ± SE. Multiple group comparisons were made by analysis of variance (F-test) with Bonferroni-adjusted t-tests and paired or unpaired t-test was used, as appropriate, for two-group comparisons. A two-tailed *p* < 0.05 was taken to indicate a statistically significant difference. Nonlinear least square curve fitting was performed with Clampfit in pClamp 9.0 or GraphPad Prism 5.0.

## Results

### Defective Trafficking of h-ERG after Chronic Oxidative Stress

**Figure 1:**
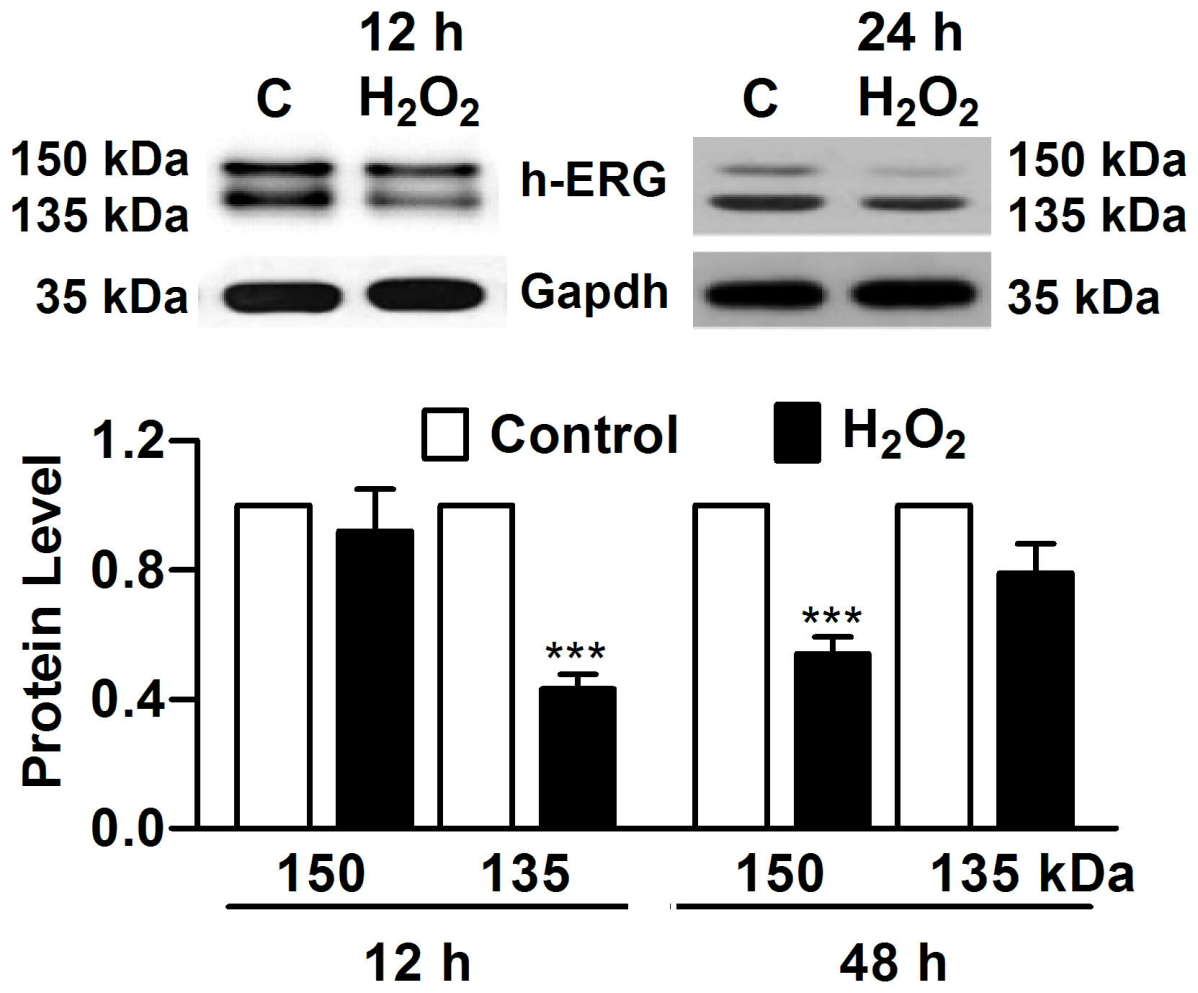
Defective trafficking of h-ERG after chronic oxidative stress in HEK293 cells stably expressing the h-ERG gene. Cells were incubated with H_2_O_2_ (40 nmol/L) and h-ERG protein was detected using Western blot analysis at 12 h and 48 h after oxidative stress. H-ERG protein appeared ass double bands with the lower band (135 kDa) representing the immature core glycosylated protein situated in endoplasmic reticulum (ER) and the higher band (155 kDa) for the mature fully glycosylated h-ERG being incorporated into the cytoplasmic membrane. C: Control. ***P<0.001 H_2_O_2_ *vs* Control; n=6.

Immunoblotting revealed double bands of h-ERG protein, in HEK293 cells with stable h-ERG expression, with 135 kDa representing the immature core glycosylated protein situated in the cytoplasm (presumably in ER and Golgi) and the 155 kDa band for the mature fully glycosylated h-ERG being incorporated into the cytoplasmic membrane. Twelve hours after H_2_O_2_ treatment in h-ERG-expressing HEK293 cells, the total h-ERG protein level was significantly reduced, as indicated by the decreased sum of 135 and 150 kDa bands (Figure 1). By comparison, the membrane h-ERG (150 kDa) was only slightly reduced, relative to the greater extend of reduction of the cytosolic h-ERG (135 kDa), indicating that h-ERG was primarily downregulated in its expression at this stage. Intriguingly, with 48 h oxidative stress, the membrane h-ERG became substantially decreased despite that the cytosolic h-ERG was decreased to a lesser degree (Figure 1), indicating that the trafficking deficiency became predominant relative to expression downregulation of h-ERG. 

Similar H_2_O_2_-induced trafficking impairment of r-ERG (rat ERG) was consistently observed in neonatal rat ventricular myocytes (NRVMs; Figure S1 in File S1) [35].

### Reciprocal Changes of MiR-17-5p and ER Stress-Related Chaperones after Chronic Oxidative Stress

**Figure 2:**
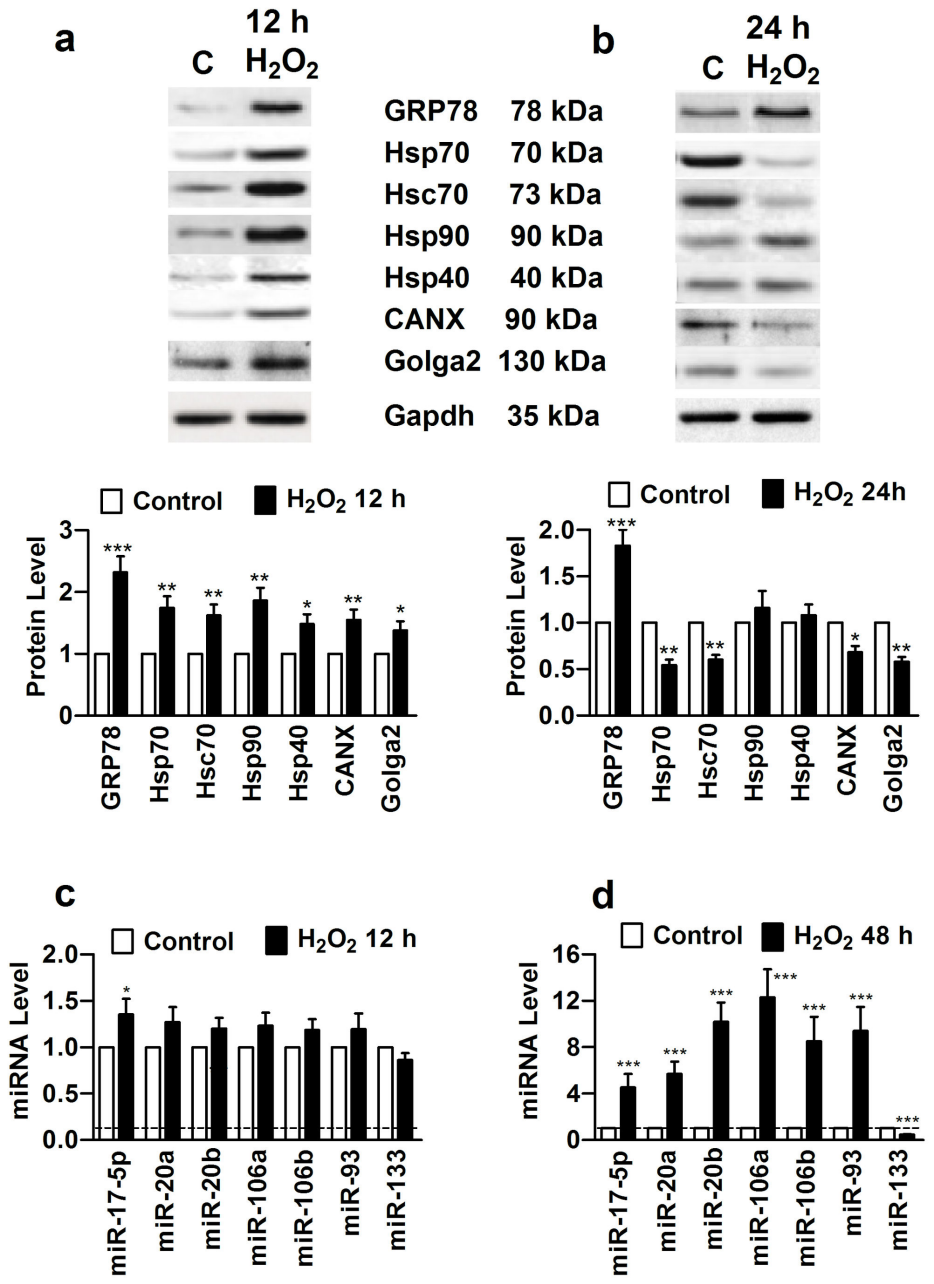
Reciprocal changes of expression between miR-17-5p and ER stress-related chaperones after chronic oxidative stress in h-ERG-expressing HEK293 cells. Cells were incubated with H_2_O_2_ (40 nmol/L) for at 12 h and 48 h. (a) & (b) Expression of ER stress-related chaperones at 12 and 48 h after oxidative stress, respectively, by Western blot analysis. *P<0.05, **P<0.01 & ***P<0.001 H_2_O_2_ *vs* Control; n=6; (c) & (d) Expression of miR-17-5p seed family miRNAs, at 12 and 48 h after oxidative stress, respectively, using real-time RT-PCR. ***P<0.001 H_2_O_2_ *vs* Control; n=6. Similar results were observed in neonatal rat ventricular myocytes (Figure S7 in File S1).

To investigate whether the development of h-ERG trafficking impairment after chronic oxidative stress is related to ER stress and its related chaperones, expression of some important chaperones was determined in both HEK293 cells and NRVMs. With 12 h H_2_O_2_ insult, GRP78 protein was markedly upregulated in HEK293 cells (Figure 2a), a hallmark of ER stress, along with upregulation of Hsp70, Hsc70, Hsp90, Hsp40, CANX, and Golga2. However, following continuous 48 h H_2_O_2_ treatment, Hsp70, Hsc70, CANX, and Golga2 were found substantially downregulated (Figure 2b). 

To see if this pattern of expression alterations of the chaperones is related to miRNA regulation, we first conducted computational prediction of miRNA targeting of these chaperones using Targetscan online site. We unexpectedly identified miR-17-5p, along with other miRNAs (miR-20a and b, miR-106a and b, and miR-93) sharing the same seed motif as miR-17-5p, as a potential common regulator of Hsp70, Hsc70, CANX, and Golga2 (Figures S2-S5 in File S1). This prompted us to exploit the relationships between miR-17-5p and the chaperones. We began with expression of miR-17-5p and other members of this seed family in HEK293 cells, using quantitative real-time RT-PCR. We consistently observed robust upregulation of these miRNAs 48 h after H_2_O_2_ treatment (Figure 2c). Most intriguingly, these miRNAs were slightly downregulated in an earlier stage of ER stress, 12 h following oxidative stress. As a parallel control, miR-133 was initially upregulated 12 h after H_2_O_2_ and downregulated 48 h after H_2_O_2_ (Figure 2d). These reciprocal changes of expression between the chaperones and miR-17-5p seed family members strongly suggest their targeting relationships.

Similar reciprocal changes of expression between the chaperones and miR-17-5p seed miRNAs were consistently observed in NRVMs (Figure S6 in File S1).

### MiR-17-5p Targets Multiple ER Stress-Related Chaperones

**Figure 3:**
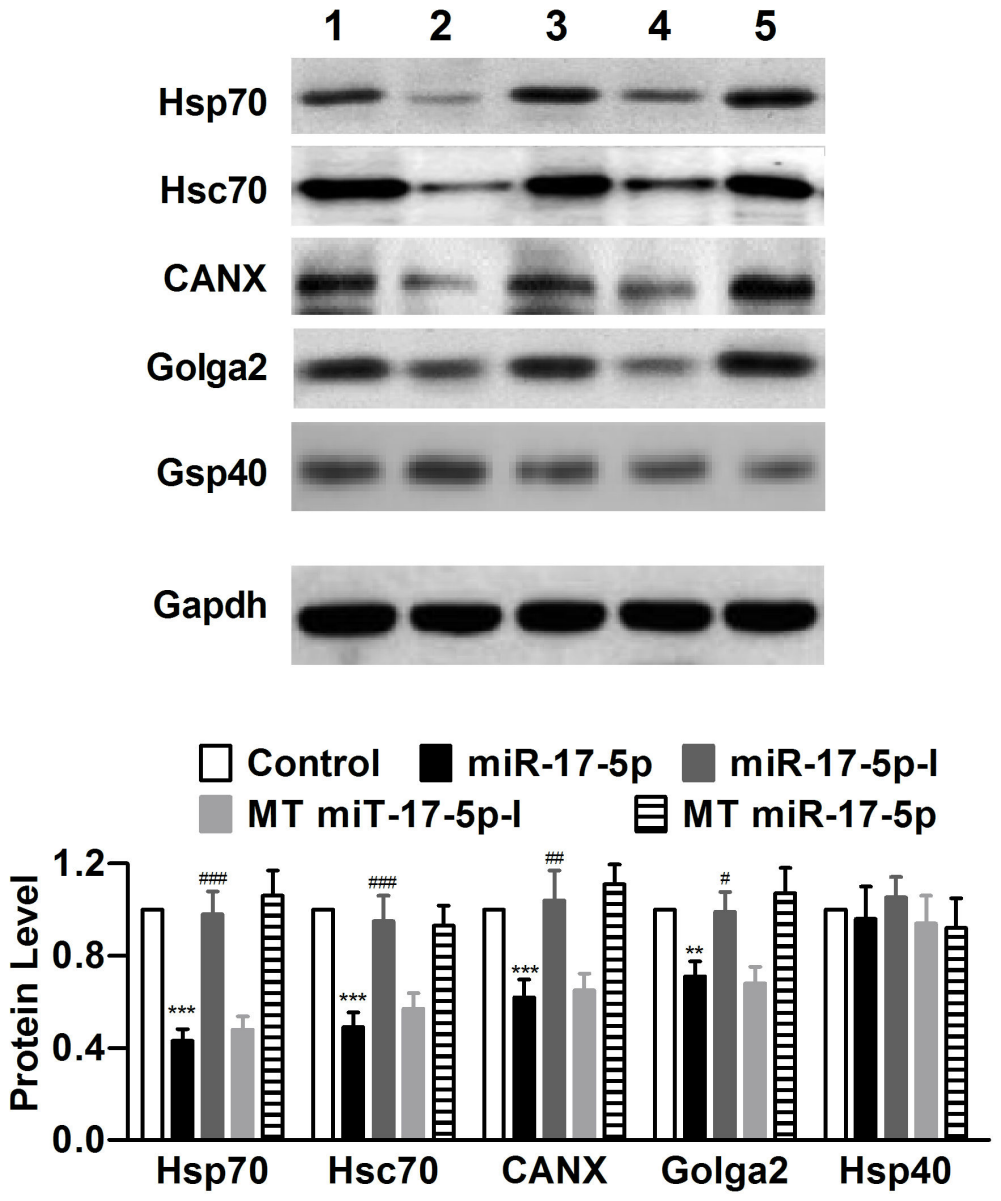
Experimental verification of Hsp70, Hsc70, CANX, and Golga2 as target genes of miR-17-5p by Western blot analysis in neonatal rat ventricular myocytes (NRVMs). miR-17-5p-I: miR-17-5p inhibitor (it was co-transfected with miR-17-5p mimic); MT miR-17-5p: mutant miR-17-5p; MT miR-17-5p-I: mutant miR-17-5p inhibitor. Hsp40 was used as a negative control for the target chaperones, and MT miR-17-5p and MT miR-17-5p-I were used as negative controls for miR-17-5p. ***P<0.001 miR-17-5p *vs* Control; n=6; ^#^ *P*<0.05, ^##^ *P*<0.01 & ^###^ *P*<0.001 miR-17-5p-I *vs* miR-17-5p.

To experimentally establish the chaperones as cognate target genes for miR-17-5p, we treated cells h-ERG-expressing HEK293 cells with miR-17-5p mimic. As depicted in Figure 3, transfection of miR-17-5p reduced the protein levels of Hsp70, Hsc70, CANX, and Golga2, but not that of Hsp40 as a negative control. The mutant miR-17-5p did not produce any significant alterations of these proteins. The downregulation caused by miR-17-5p was mitigated with co-transfection of miR-17-5p inhibitor (miR-17-5p-I), and mutant miR-17-5p-I failed to do so. 

**Figure 4:**
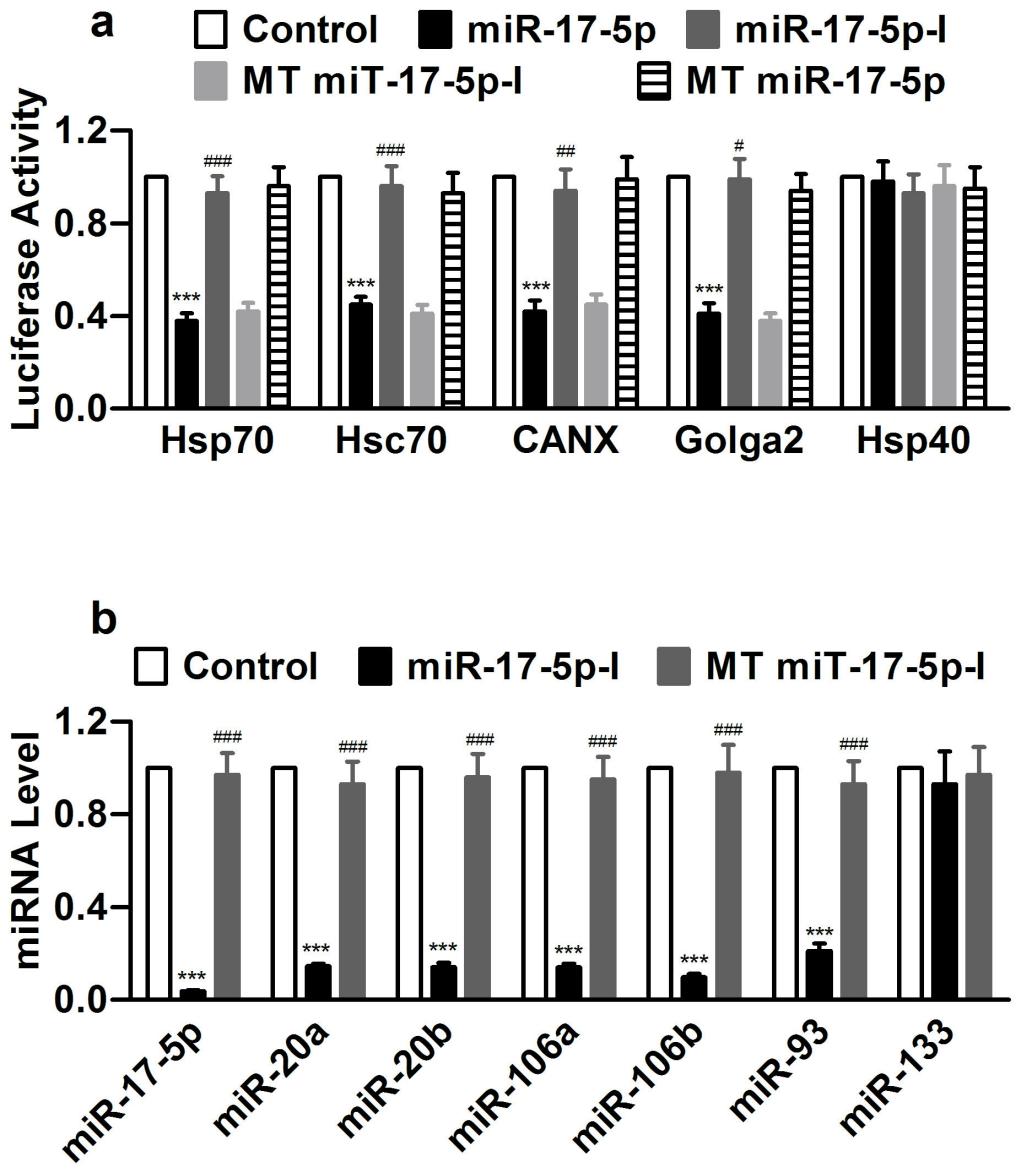
Experimental verification of Hsp70, Hsc70, CANX, and Golga2 as target genes of miR-17-5p by luciferase reporter gene activity assay in HEK293 cells. (a) Evaluation of effects of miR-17-5p on luciferase activities elicited by chimeric 3’UTR—luciferase vector to verify the interaction between miR-17-5p and the target sites of the ER stress-related chaperones. miR-17-5p-I: miR-17-5p inhibitor (co-transfected with miR-17-5p mimic); MT miR-17-5p: mutant miR-17-5p; MT miR-17-5p-I: mutant miR-17-5p inhibitor. Hsp40 was used as a negative control for the target chaperones, and MT miR-17-5p and MT miR-17-5p-I were used as negative controls for miR-17-5p. ***P<0.001 miR-17-5p *vs* Control; n=6; ^#^ *P*<0.05, ^##^ *P*<0.01 & ^###^ *P*<0.001 miR-17-5p-I *vs* miR-17-5p. (b) Real-time RT-PCR quantification to verify the efficacy of miR-17-5p-I to knock down miR-17-5p seed miRNAs. miR-133 was used as a negative control. ***P<0.001 miR-17-5p *vs* Control; n=6; ^#^ *P*<0.05, ^##^ *P*<0.01 & ^###^ *P*<0.001 miR-17-5p-I *vs* miR-17-5p.

We subsequently verified the functional interactions between the miR-17-5p and the binding sites in the 3’UTRs of the chaperones, using luciferase reporter gene assay. Figure 4a shows substantial reduction of luciferase activities in cells transfected with miR-17-5p, an effect reversed by its antisense inhibitor. As expected, the mutant miR-17-5p had no effects on luciferase activities, and the mutant inhibitor did not alter the effects of miR-17-5p. 

We then verified the ability of miR-17-5p-I to knockdown miR-17-5p (Figure 4b). As expected, this miR-17-5p-I also decreased the levels of other miR-17-5p seed family miRNAs, as they share high sequence homology (Figure S7 in File S1).

### MiR-17-5p Impairs h-ERG Trafficking

**Figure 5:**
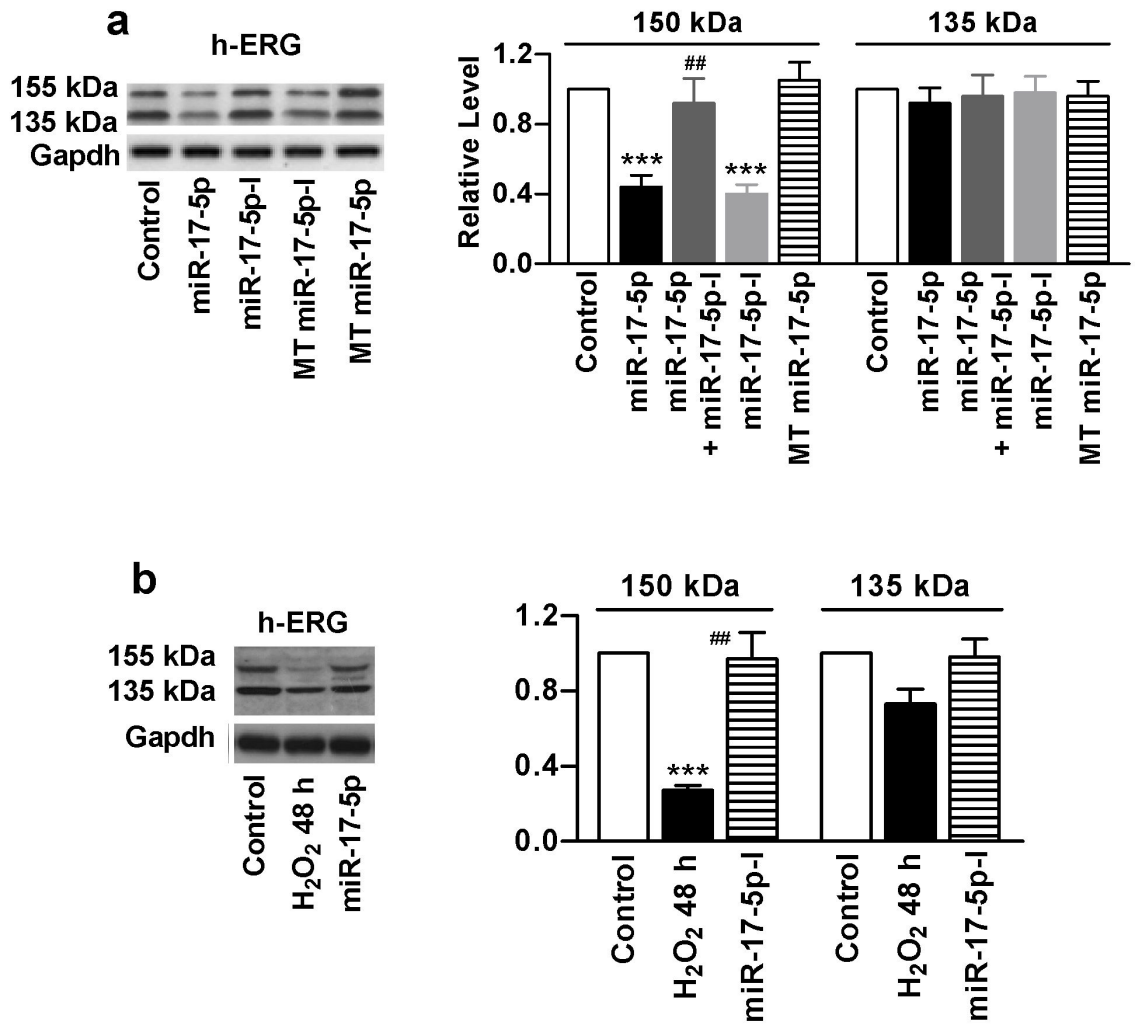
Direct effects of miR-17-5p on h-ERG trafficking in h-ERG-expressing HEK293 cells. (a) Effects of exogenously applied miR-17-5p mimic on h-ERG trafficking assessed by Western blot analysis. miR-17-5p-I: miR-17-5p inhibitor (co-transfected with miR-17-5p mimic); MT miR-17-5p: mutant miR-17-5p. ***P<0.001 miR-17-5p *vs* Control; ^###^ *P*<0.001 miR-17-5p-I *vs* miR-17-5p; n=6; (b) Effects of miR-17-5p-I on oxidative stress-induced trafficking disruption of h-ERG. ***P<0.001 H_2_O_2_ *vs* Control; ^###^ *P*<0.001 miR-17-5p-I *vs* H_2_O_2_; n=5.

Transfection of miR-17-5p in h-ERG-expressing HEK293 cells reduced the upper band (150 kDa) indicating the defective trafficking without altering the lower band for the total expression level of h-ERG proteins (Figure 5a). Co-application of miR-17-5p-I rescued the trafficking disturbance. The mutant miR-17-5p failed to affect h-ERG trafficking and the mutant miR-17-5p-I was unable to affect the rescuing effect of miR-17-5p-I.

 Furthermore, in cells exposed to H_2_O_2_ for 48 h and concordantly treated with miR-17-5p-I, the impairment of h-ERG protein was remarkably relieved (Figure 5b). This effect was not seen in cells treated with the mutant miR-17-5p-I.

**Figure 6:**
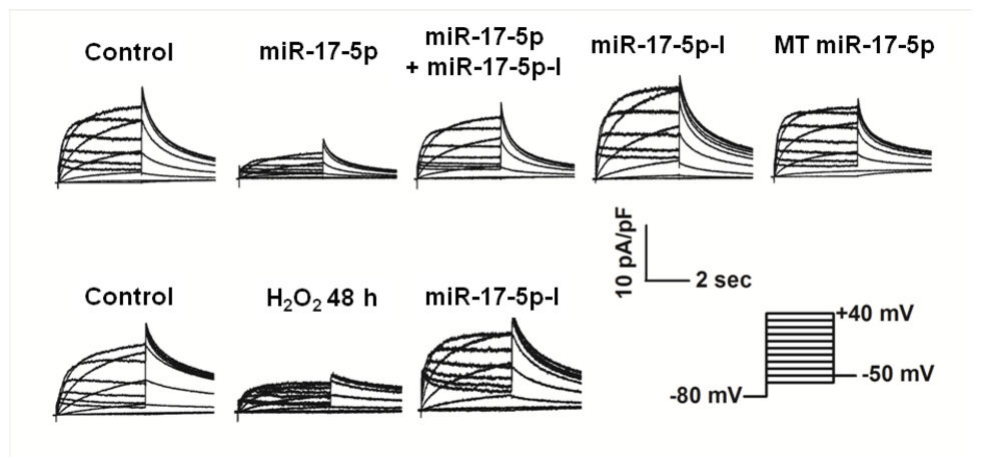
Effects of miR-17-5p on h-ERG current (I_h-ERG_) recorded using whole-cell patch-clamp techniques. The voltage protocol was shown in the inset. miR-17-5p-I: miR-17-5p inhibitor (co-transfected with miR-17-5p mimic); MT miR-17-5p: mutant miR-17-5p. Similar results were observed in another 7 cells for each case.

 Finally, our whole-cell patch-clamp recordings demonstrated that transfection of miR-17-5p suppressed h-ERG current (I_h-ERG_) in HEK293 cell with stable h-ERG expression, an effect reversed by co-transfection of miR-17-5p-I, but not by the mutant inhibitor (Figure 6). Moreover, treatment with H_2_O_2_ for 48 h substantially reduced I_h-ERG_, and this effect was eliminated by miR-17-5p-I, indicating the role of miR-17-5p in suppressing the current. 

### AP1 Activation Underlies MiR-17-5p Upregulation and H-ERG Trafficking Impairment

MiR-17-5p belongs to the miR-17~92 cluster, located on the human chromosome 13q31, and is a prototypical example of a polycistronic miRNA gene encoding six miRNAs (miR-17-5p, miR-18, miR-19a, miR-19b, miR-20 and miR-92). This cluster has two paralogs, miR-106a~363 (miR-106a, miR-18b, miR-19b-2, miR-20b, miR-92a-2 and miR-363, located on the X chromosome) and miR-106b~25 (miR-106b, miR-93, and miR-25, located on human chromosome 7), which are located on different chromosomes but contain individual miRNAs that are highly similar to those encoded by the miR-17~92 cluster [36,37]. To understand how the miR-17-5p seed family miRNAs belonging to different clusters were simultaneously upregulated after prolonged period of oxidative stress, we analyzed the promoter regions of these three clusters. The human miR-17~92 cluster is located in the third intron of a ~7 kb primary transcript known as C13orf25 or MIR17HG1, a non-coding RNA. The miR-106b~25 cluster is located within the 13th intron of the MCM7 gene encoding DNA replication licensing factor. The miR-106a~363 cluster is an intergenic gene. Our computational analysis predicted AP1 (c-jun/c-fos), a well-known transactivator of many genes, as a common regulator of transcription of the miR-17~92 cluster and its two paralogs. AP1 has been shown to cause apoptosis in ischemic myocardium and other conditions associated with oxidative stress as the transcriptional activity of AP1 is redox-sensitive through the conserved cysteine residues located in the DNA-binding domain [38-41]. 

**Figure 7:**
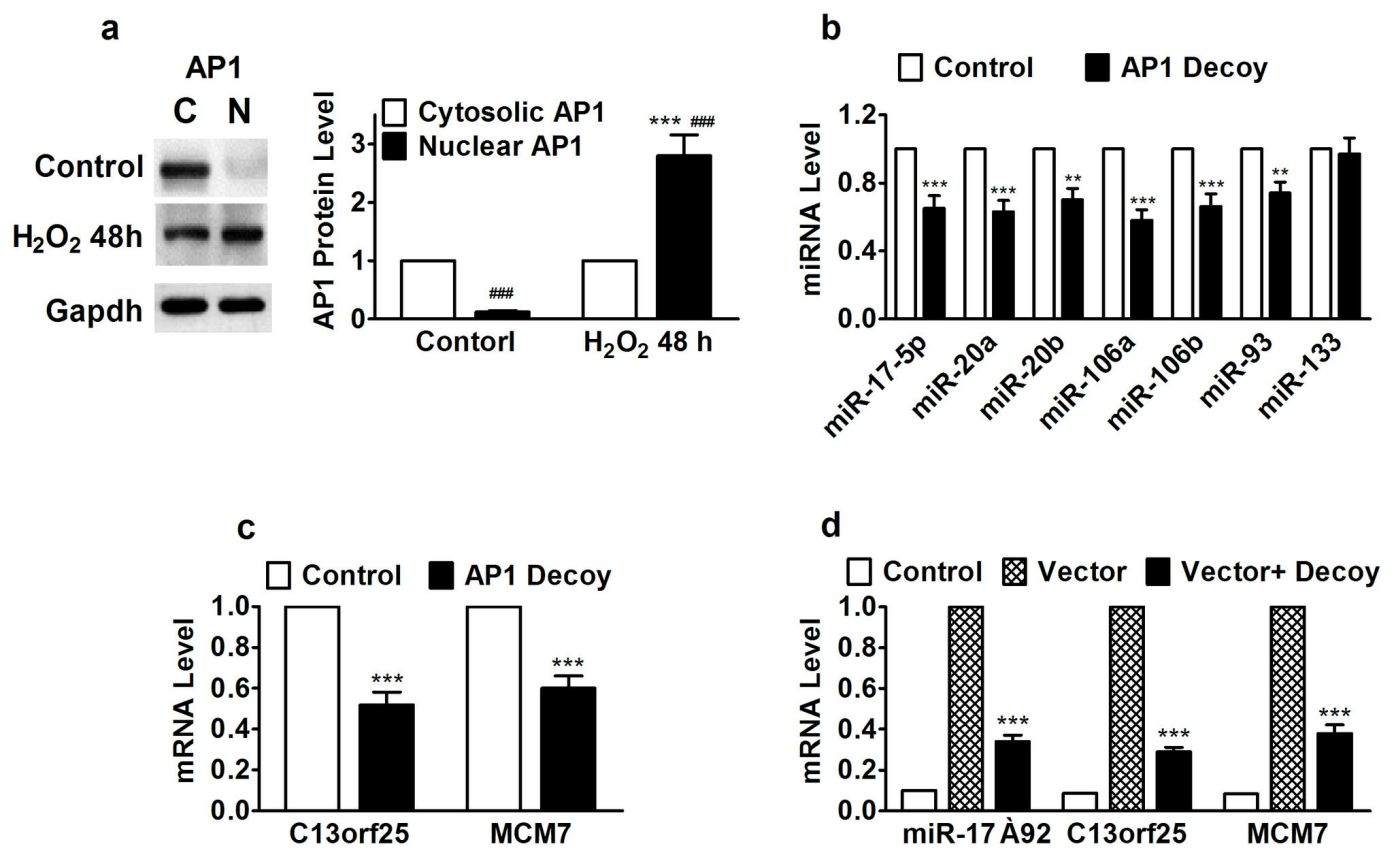
Effects of activating protein-1 (AP1) on transcriptional stimulation of the miR-17-5p seed miRNAs belonging to miR-17~92, miR-106a~363, and miR-106b~25 clusters in HEK293 cells. (a) Nuclear translocation of AP1 after chronic H_2_O_2_ treatment, assessed by Western blot analysis of cytosolic and nuclear AP1 protein levels. ***P<0.001 H_2_O_2_ *vs* Control; ^###^ *P*<0.001 Nuclear *vs* Cytosolic; n=5; (b) Effects of AP1 decoy to sequestrate AP1 on expression of miR-17-5p seed miRNAs. **P<0.01 & ***P<0.001 H_2_O_2_ *vs* Control; n=5; (c) Effects of AP1 decoy to sequestrate AP1 on expression of C13orf25 and MCM7, the host genes of miR-106a~363 cluster and miR-106b~25 cluster, respectively. **P<0.01 & ***P<0.001 H_2_O_2_ *vs* Control; n=5; (d) Effects AP1 decoy to on luciferase activities driven by luciferase vector containing the promoter regions of miR-17~92, C13orf25, and MCM7. ***P<0.001 AP1 Decoy *vs* Vector alone; n=6.

To explore the possible role of AP1 in the concurrent upregulation of the miR-17~92 cluster and its two paralogs, we first confirmed the nuclear translocation of AP1 in cells exposed to H_2_O_2_ (Figure 7a). We then applied the decoy oligodeoxynucleotide fragment against AP1 to NVRCs to sequestrate AP1 in these cells. QRT-PCR confirmed the downregulation of the miR-17-5p seed family miRNAs (Figure 7b), along with C13orf25 and MCM7 (Figure 7c), the host genes for the miR-17~92 cluster and the miR-106b~25 cluster, respectively. Next, we employed the luciferase reporter gene assay to measure the transcriptional activities of AP1 on the expression of the miR-17-5p seed family members. Our data demonstrated the ability of AP1 decoy to mitigate the luciferase activities driven by the promoters of the miR-17~92 cluster and its two paralogs (Figure 7d). 

**Figure 8:**
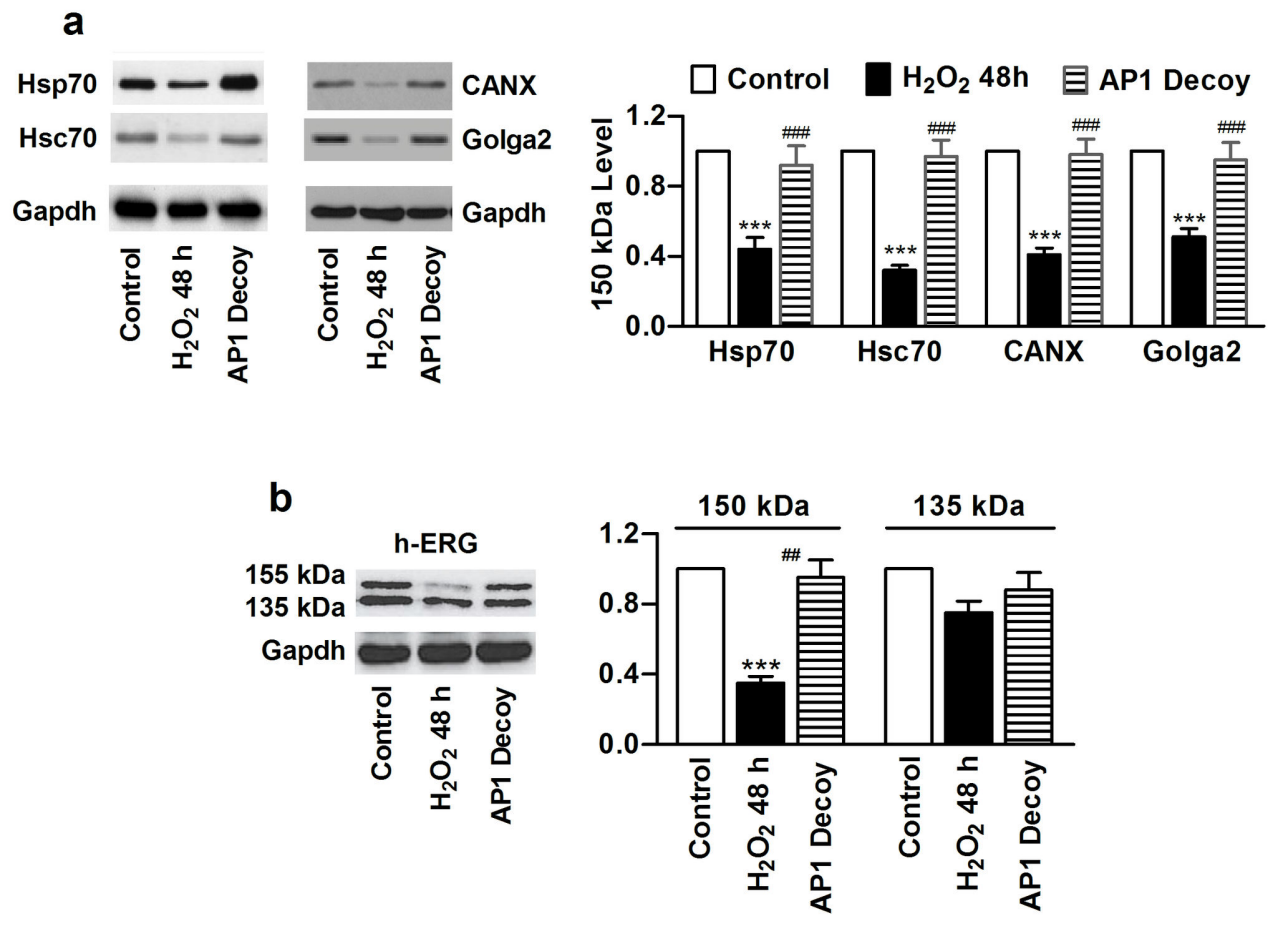
Effects of activating protein-1 (AP1) on expression of chaperones and trafficking of h-ERG in HEK293 cells. (a) Abolishment of chronic H_2_O_2_-induced downregulation of chaperones by AP1 decoy oligodeoxynucleotides, determined by Western blot analysis. ***P<0.001 H_2_O_2_ *vs* Control; ^###^ *P*<0.001 AP1 Decoy *vs* H_2_O_2_; n=4; (b) Correction of h-ERG trafficking impairment induced by chronic H_2_O_2_ exposure by AP1 decoy. ***P<0.001 H_2_O_2_ *vs* Control; ^##^ *P*<0.01 AP1 Decoy *vs* H_2_O_2_; n=4.

Most notably, application of AP1 decoy reversed the downregulation of Hsp70, Hsc70, CANX, and Golga2 induced by H_2_O_2_ (Figure 8a). Meanwhile, AP1 decoy was able to rescue h-ERG channel proteins from trafficking deficiency in the presence of oxidative stress (Figure 8b).

## Discussion

In the present study, we investigated the role of miR-17-5p in regulating trafficking of human ether-a-go-go-related gene (h-ERG) K^+^ channel under the condition of chronic oxidative stress. The results revealed that (1) h-ERG trafficking was impaired after prolonged period of oxidative stress; (2) chronic oxidative stress induced reciprocal changes of expression between miR-17-5p, along with several other miRNAs sharing the identical seed motif, and the ER stress-related chaperones (Hsp70/Hsc70, CANX, and Galga2), with the former upregulated and the latter downregulated; (3) miR-17-5p targets all these ER stress-related chaperones to cause defective trafficking of h-ERG; (4) Either upregulation of endogenous or application of exogenous miR-17-5p reduced h-ERG current density, and the effects were abolished by miR-17-5p inhibitor; and (5) sequestration of AP1 eliminated the upregulation of miR-17-5p and other members of the seed family, and meanwhile ameliorated impairment of h-ERG trafficking. These findings allowed us to reach the conclusion that deregulation of the miR-17-5p seed family miRNAs can cause severe impairment of h-ERG trafficking through targeting multiple ER stress-related chaperones, and activation of AP1 likely accounts for the deleterious upregulation of these miRNAs, after prolonged oxidative stress. We therefore proposed the following signaling pathway: chronic oxidative stress ― ER stress ― activation/upregulation of AP1 ― upregulation of miR-17-5p/other seed members ― repression of ER stress-related chaperones ― defective trafficking of h-ERG ― h-ERG current reduction ― QT prolongation. 

H-ERG is an α-subunit of a K^+^ channel for rapid delayed rectifier current (*I*_Kr_), the major repolarizing current in cardiac cells. H-ERG is the major cause of genetic and drug-induced long QT syndromes, the most common mechanism of h-ERG channel dysfunction in long QT syndromes is defective trafficking of h-ERG protein to the cell surface. Recent studies have shown that oxidative stress causes functional impairment of h-ERG with evidence for defective trafficking of h-ERG protein contributing to acquired LQTS, but the mechanisms are yet to be determined. H-ERG trafficking is a complex process involving a number of chaperones and co-chaperones. After synthesized in ER, h-ERG protein undergoes sequential modifications in ER and Golgi, before being inserted into cytoplasmic membrane. The primary modification is N-terminal glycosylation. Heat shock protein 70 and heat shock cognate protein 70 bind to the immature core-glycosylated h-ERG, followed by binding of calnexin; these chaperones work together to add polysarcharides to the channel protein. Then h-ERG is transported to golgi where it is further processed by Golga2 protein for maturation to have right folding for insertion into the cytoplasmic membrane. In an earlier study reported by Ficker et al [16], Hsp90 and Hsp70 were found to interact directly with the core-glycosylated form of h-ERG WT present in the endoplasmic reticulum but not the fully glycosylated, cell-surface form, indicating that Hsp90 and Hsp70 are crucial for the maturation of h-ERG. A recent study generated evidence in support of the role of Hsp90 in h-ERG trafficking [15]. In addition to Hsp70, heat-shock cognate protein 70 (Hsc70) has also been documented to be involved in h-ERG trafficking [14]. The interaction of h-ERG channels with the ER marker/chaperone protein calnexin (CANX) has also been reported [17,18]. The study by Delisle et al [13] demonstrated that small GTPase controls the Golgi processing and plasmalemmal expression of h-ERG K^+^ channels. In addition, FKBP38 as a co-chaperone of h-ERG was found to contribute via the Hsc70/Hsp90 chaperone system to the trafficking of wild type and mutant h-ERG proteins [11]. Hsp40 chaperones were reported to reduce h-ERG trafficking efficiency by promoting degradation of the proteins [42]. These results indicate that h-ERG trafficking is under the control by a variety of chaperones and co-chaperones, and dysfunction of any of these proteins can render functional impairment of h-ERG. Our data in this study provided evidence in support of this notion. Moreover, our findings unraveled the pathological trafficking disturbance of h-ERG in response to oxidative stress that has been shown to be a primary detrimental factor to many diseased conditions of the heart such as myocardial ischemia/reperfusion injury and heat failure [43-45]. This trafficking impairment may contribute to the abnormal QT prolongation and the associated arrhythmogenesis under these pathological settings [46,47]. 

Strikingly, the chaperones Hsp70/Hsc70 and CANX that had been documented to be regulators of h-ERG trafficking and Galga2 were all found to be target genes for miR-17-5p in this study. There was reciprocal relationship between miR-17-5p and these chaperones in terms of their expression after long-term treatment of H_2_O_2_. And this reciprocal change of expression between the chaperones and miR-17-5p occurred only after prolonged period (48 h) of oxidative stress. During the early phase of H_2_O_2_ insult (12 h), the chaperones were upregulated and miR-17-5p remained unaltered. Application of miR-17-5p mimic, but not the mutant construct, decreased the levels of these proteins, which was readily reversed by the specific inhibitor of this miRNA. Luciferase reporter gene assay further confirmed the interactions between miR-17-5p and its seed sites in the 3’UTR of the genes encoding Hsp70/Hsc70, CANX, and Golga2. This targeting relationship likely underlies at least partially the downregulation of these chaperones in the presence of oxidative insult. And downregulation of these proteins is deemed to cause severe defective trafficking of h-ERG. More notable is the finding that in addition to miR-17-5p, other miRNAs with identical seed motif as miR-17-5p were also upregulated after prolonged exposure to H_2_O_2_. This aberrant upregulation of the miR-17-5p seed family miRNAs is expected to produce powerful repression of their target genes. However, since these miRNAs act on the same seed sequence presumably by competitive binding to the site, synergetic or additive effects may not be expected. Thus, functionally, they may act like one miRNA. Indeed, in our experiments, though we were primarily focused on miR-17-5p alone, the antisense inhibitor knocked down all members of the miR-17-5p seed family, as they share tremendous sequence homology (Figure S7 in File S1). This result is also consistent with previous studies showing inhibition of seed family miRNAs by a single antisense [48,49]. Most intriguingly, a recent study showed that miR-17-5p regulates endocytic trafficking through targeting four trafficking-related proteins (TBC1D2, M6PR, ASAP2 and LDLR) in HeLa cells [50]. It would be interesting to know whether these trafficking-related proteins also play a role in h-ERG channel protein in future studies. This study plus the present study indicate that miR-17-5p is a trafficking protein regulator miRNA. Furthermore, our study provided preliminary evidence for the role of AP1 in mediating oxidative stress-induced deregulation of miR-17-5p seed family miRNAs: inhibition of AP1 prevented upregulation of these miRNAs and consequently the downregulation of the chaperones was mitigated. Particularly notable is that sequestration of AP1 by its decoy molecule rescued the defective trafficking of h-ERG proteins. The transcriptional activity of AP1 is known to be redox-sensitive and is boosted up in ischemic myocardium and other conditions associated with oxidative stress [38-41]. While previous studies have concentrated on the role of AP1 in cardiomyocyte apoptosis in response to oxidative stress, the present study unraveled a novel cellular function of this transcription factor: impairing h-ERG trafficking. This finding allows us to have a full picture about how chronic oxidative insult induces defective trafficking of h-ERG channel proteins. It appears plausible that oxidative stress triggers AP1 activation, which then transcriptionally activates all three miRNA clusters harboring miR-17-5p seed miRNAs. Upregulation of miR-17-5p in turn downregulates the related chaperones to reduce h-ERG trafficking leading to associated electrical disturbance of the heart. However, more detailed experimentations are required to confirm this signaling pathway. 

### Study Limitations

One important limitation of the present study is that the main body of the experiments was conducted in h-ERG-expressing HEK293 cells, in order to have a discrete measurement of h-ERG protein and current. Though it is widely used model for studying h-ERG trafficking, the cellular context of HEK293 may not identical to cardiac cells. Nonetheless, our data showing qualitatively the same changes of miR-17-5p (and other seed members) and the chaperones (Hsp70/Hsc70, CANX, and Golga2) would suggest that cardiomyocytes and HEK293 cells have similar response to oxidative stress. Another limitation of the study is that our study was carried out under in vitro conditions, and the results from such cellular model may not reflect the true pathological process of the heart. Not until further studies under in vivo conditions to verify the findings can our data have valid applicability.

## Conclusions

Collectively, we show here that chronic oxidative stress caused upregulation of the miR-17-5p seed family miRNAs, which in turn target the genes encoding the ER stress-related chaperones to downregulate these proteins, leading to defective trafficking and functional impairment of h-ERG channel proteins. Activation of AP1 by oxidative stress may be an upstream molecular mechanism for the upregulation of miR-17-5p. These findings provide a link between miRNAs and h-ERG trafficking disturbance in the presence of oxidative stress and this link may contribute to the electrical disturbance in the heart under the conditions associated with oxidative stress. Hence, miR-17-5p seed family miRNAs may be considered a new target for antiarrhythmic therapy. Moreover, our data also represent, to our knowledge, the first to show the regulation of ion channels by miRNAs through modulating trafficking process and therefore revealed a novel cellular function of miRNAs.

## Supporting Information

File S1 **Supporting Figures.**
(DOC)
