# Supplementary material for: MiR-17-5p Impairs Trafficking of H-ERG K+ Channel Protein by Targeting Multiple ER Stress-Related Chaperones during Chronic Oxidative Stress
Source: PLoS One. 2013 Dec 30;8(12):e84984. doi: 10.1371/journal.pone.0084984 (PMC3875566; doi:10.1371/journal.pone.0084984)
Supplement: File S1 — Supporting Figures. (DOC) [file pone.0084984.s001.doc]

**Supplementary Materials for:**

**MiR-17-5p Impairs Trafficking of H-ERG K^+^ Channel Protein by Targeting Multiple ER Stress-Related Chaperones during Chronic Oxidative Stress**

**Qi Wang, Weina Hu, Mingming Lei, Yong Wang, Bing Yan, Jun Liu, Ren Zhang,**

**Yuanzhe Jin***

The Fourth Affiliated Hospital of China Medical University, Shenyang, Liaoning Province, P. R. China

Running Title: **Regulation of h-ERG trafficking by miRNAs**

**Correspondence to:**

*E-mail: [cmujyz@gmail.com](mailto:cmujyz@gmail.com)

**Figure S1**

**Fig. S1.** Defective trafficking of r-ERG after chronic oxidative stress in neonatal rat ventricular myocytes (NRVMs). Cells were incubated with H_2_O_2_ (40 nmol/L) and r-ERG protein was detected using Western blot analysis at 12 h and 48 h after oxidative stress. R-ERG protein appeared ass double bands with the lower band (135 kDa) representing the immature core glycosylated protein situated in endoplasmic reticulum (ER) and the higher band (155 kDa) for the mature fully glycosylated r-ERG being incorporated into the cytoplasmic membrane. C: Control. ****P*<0.001 H_2_O_2_ *vs* Control; n=5.

Figure S2

**Fig. S3.** Alignment between miR-17-5p seed miRNAs and the binding sites in the 3’UTR of Hsp70. “:” indicates wobble pairing.

Figure S3

**Fig. S4.** Alignment between miR-17-5p seed miRNAs and the binding sites in the 3’UTR of Hsc70. “:” indicates wobble pairing.

Figure S4


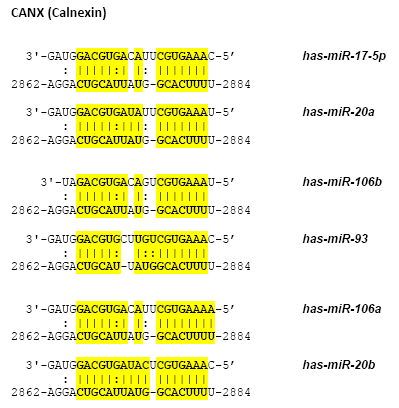


**Fig. S5.** Alignment between miR-17-5p seed miRNAs and the binding sites in the 3’UTR of CANX. “:” indicates wobble pairing.

Figure S5

**Fig. S6.** Alignment between miR-17-5p seed miRNAs and the binding sites in the 3’UTR of Golga2. “:” indicates wobble pairing.

Figure S6

**Fig. S6.** Reciprocal changes of miR-17-5p and ER stress-related chaperones after chronic oxidative stress in NRVMs. Cells were incubated with H_2_O_2_ (40 nmol/L) for at 12 h and 48 h. (a) & (b) Expression of ER stress-related chaperones at 12 and 48 h after oxidative stress, respectively, by Western blot analysis. **P*<0.05, ***P*<0.01 & ****P*<0.001 H_2_O_2_ *vs* Control; n=6; (c) & (d) Expression of miR-17-5p seed family miRNAs, at 12 and 48 h after oxidative stress, respectively, using real-time RT-PCR. ****P*<0.001 H_2_O_2_ *vs* Control; n=5.

Figure S7

**Fig. S7.** Alignment of mature miR-17-5p seed miRNAs showing the identical seed motif and overall high conservation of sequence.
